# Remnant-cholesterol-inflammatory-index as a novel predictor of cardiometabolic risk and cardiovascular mortality in elderly patients with severe aortic stenosis

**DOI:** 10.3389/fendo.2026.1894558

**Published:** 2026-10-19

**Authors:** Annamaria Mazzone, Melania Gaggini, Ilenia Foffa, Serena Del Turco, Fabrizio Minichilli, Sergio Berti, Cristina Vassalle

**Affiliations:** 1 Diagnostic and Interventional Cardiology Department, Fondazione Toscana Gabriele Monasterio, Massa, Italy; 2 Italian National Research Council, Institute of Clinical Physiology, Pisa, Italy; 3 Laboratory Medicine Department, Fondazione Toscana Gabriele Monasterio, Pisa, Italy

**Keywords:** aortic stenosis, cardiometabolic risk, elderly, inflamm-aging, mortality, remnant cholesterol inflammatory index

## Abstract

**Background:**

Inflammaging activates a vicious cycle common to the progression of structural cardiovascular alterations, cardiometabolic diseases as well as degenerative aortic stenosis (AoS). The remnant cholesterol inflammatory index (RCII) is a new recently proposed biomarker that combines remnant cholesterol and high-sensitivity C-reactive protein, resembling inflammaging. However, the role of RCII in terms of cardiometabolic risk and cardiovascular mortality in AoS elderly patients has not been evaluated still now.

**Aim:**

to assess the association between RCII and cardiovascular risk factors and mortality in AoS elderly patients.

**Results:**

RCII levels correlated with systemic inflammation biomarkers (closer correlation with interleukin-6), glucose, creatinine, vascular endothelial growth factor and frailty in AoS patients. Inverse correlations were observed with albumin (typically reduced in inflammatory states) and with HDL concentration. The Kaplan-Meier survival estimates showed significantly worse survival in patients with elevated RCII levels (p<0.01) (mean follow-up 19 ± 4 months). In an exploratory multivariable Cox analysis, higher RCII was associated with cardiovascular mortality (HR = 3.5, 95% CI: 1.0–12.0).

**Conclusion:**

RCII is associated with cardiometabolic risk and adverse prognosis in elderly patients with AoS, providing an additional perspective on the prognostic implications of the coexistence of lipid abnormalities and low-grade inflammation in this population. However, the limited number of cardiovascular deaths warrants cautious interpretation of this estimate. Moreover, external validation, standardized thresholds, serial measurements, and comparison with established risk models are needed before RCII clinical implementation.

## 1 Introduction

Aortic stenosis (AoS) is not a passive condition elicited by calcium deposition in association with aging, but instead a process characterized by the activation of metabolic and inflammatory processes, closely associated with inflammaging (the age-related increase of chronic, low-grade systemic inflammation) and metabolic dysfunction (1, 2). In this context, the same inflammatory and metabolic protagonists which characterized the development of the atherosclerotic plaque (e.g. obesity, Type 2 diabetes-T2D, oxidized lipids, T lymphocytes, and monocytes) are also important in the events that contribute to valvular fibrosis and calcification processes (3, 4). During ageing, chronic sterile low-grade inflammation - called inflammaging - develops, which contributes to the pathogenesis of age-related diseases. Inflammaging in particular feeds a vicious cycle favoring the progression of structural cardiovascular (CV) alterations, cardiometabolic diseases as well as degenerative AoS (3, 4).

The Remnant Cholesterol Inflammatory Index (RCII) is a biomarker proposed very recently obtained by the integration of remnant cholesterol (RC, the cholesterol contained in triglyceride-rich lipoproteins) and C-reactive protein (CRP), used to evaluate the impact of co-exposure to residual cholesterol and systemic inflammation on CV, metabolic and inflammatory risk (5, 6). Indeed, lipids, major constituents of cell membranes, sources of energy, and key determinants of a number of intercellular and intracellular processes, are mediators implicated in all phases of inflammation (7). Thus, RCII may effectively mirror “inflamma-aging” by linking lipid alterations (RC) with systemic inflammation (CRP) (8). Accordingly, RCII has been found closely related to a higher risk of CV disease and adverse CV outcomes (total and CV mortality, stroke) in different patient populations (e.g. T2D) (5, 9, 10). Moreover, RC alone is closely related with a higher risk, faster progression, and adverse events (all-cause mortality or aortic valve re-intervention) in patients with AoS (11–13). On the other hand, CRP is a key hallmark of inflamm-aging and related to age-related conditions (e.g. CV and neurodegenerative diseases and cancer) (14, 15) Accordingly, high CRP values (>3 mg/L) are associated with physical and cognitive function dysfunction and mortality in elderly (16, 17). Moreover, CRP is implicated in the progression of AoS and elevated CRP levels, similar to those found in atherosclerosis, are found in patients with degenerative AoS (18).

The RCII, which integrates both lipid- and inflammation-driven damage, likely amplifies the overall burden by reflecting vascular injury, glucose-lipid dysregulation, and impaired immune function. However, the role of the combined RCII parameter in terms of cardiometabolic risk and mortality in AoS elderly patients has not been evaluated still now. In fact, RCII, integrating both lipid-driven and inflammatory aspects, which contribute to the inflammatory and calcific processes within the valve leaflets, may be important in the AoS patients.

Therefore, this study aimed to evaluate the association of RCII and cardiometabolic risk factors and CV mortality in elderly patients with severe AoS.

## 2 Materials and methods

Characteristics of patients: Data from 103 elderly patients affected by a symptomatic severe calcified aortic valve stenosis (33 males; 83.5 ± 5.5 years) afferent to the outpatient clinic of the Ospedale del Cuore, Fondazione Toscana G. Monasterio (FTGM) in Massa, Italy (1 March 2016–30 March 2020), who underwent a multidisciplinary heart team outdoor evaluation (including clinical, surgical, laboratory and multidimensional geriatric assessment) for a tailored, frailty dependent, surgical, interventional or pharmacological treatment of valve disease, were retrospectively analyzed (19).

The inclusion criteria were considered as follows: (1) severe symptomatic AoS diagnosis with degenerative etiology, according to the AHA/ACC Guideline for the Management of Patients with Valvular Heart Disease (20); (2) a preserved left ventricular systolic function (3) symptomatology as dyspnea (NYHA class), angina, syncope; (4) age ≥75 years; (5) a written informed consent. Following exclusion criteria were considered: (1) acute heart failure, (2) hemodynamic shock or (3) active infection at the time of patient enrollment (to avoid the possible impact upon the levels of the biomarkers evaluated). The identification of frailty was identified by using the Phenotypic Fried criteria and categorized as pre-frail = 1–2 or frail = 3–5, because, in our cohort of elderly patients, none was classified as non-frail (21).

Arterial hypertension was defined when systolic blood pressure was >140 mmHg and/or diastolic pressure >90 mmHg or use of antihypertensive medication, T2D when fasting plasma glucose was >126 mg/dL or use of antidiabetic treatment, and dyslipidemia when total cholesterol was 200 mg/dL, or triglyceride 150 mg/dL, or current use of lipid-lowering drugs. Medical therapy included angiotensin-converting enzyme inhibitors, beta-blockers, calcium antagonists, mineralocorticoid receptor antagonist (MRA/ARA), lipid-lowering agents, antidiabetic agents, diuretics, oral anticoagulant therapy; the mean number of drugs per patient was 7 ± 2.5.

The study was approved by the local Ethics Committee (Comitato Etico di Area Vasta Nord Ovest [CEAVNO] No. 22239/2022).

Echocardiographic evaluation: Standard transthoracic echocardiographic imaging of all patients were performed following the AHA/ACC Guideline for the Management of Patients with Valvular Heart Disease (20).

Laboratory assessment: A venous blood sample was obtained following 12 h fasting in all patients. Blood parameters measured by automatic biochemical analyzers included a complete blood count, a complete lipid profile [total cholesterol (TC), triglycerides (TG), high-density lipoprotein cholesterol (HDL-C), low-density lipoprotein cholesterol (LDL-C)], creatinine, albumin, glycemia, inflammatory/oxidative stress-related biomarkers [CRP, uric acid, erythrocyte sedimentation rate (ESR), fibrinogen], cardiac biomarkers [brain natriuretic peptide, BNP]. Moreover, interleukin-6 (IL-6) and vascular endothelial growth factor (VEGF) were available in a subgroup of patients (n=23) and analyzed in 25 µL of plasma by using an integrated multi-analyte detection platform (MILLIPLEX MAP human cytokines magnetic bead panel, Millipore corporation, Billerica, MA, USA). This system is a compact, image-based multiplex analysis system to simultaneously detect up to 50 different analytes (e.g. proteins or nucleic acids) in a single well and in a short time (60 minutes), utilizing magnetic microspheres (MagPix Luminex) and a Charge-Coupled Device camera for fluorescence imaging (**Figure 1**).

**Figure 1:**
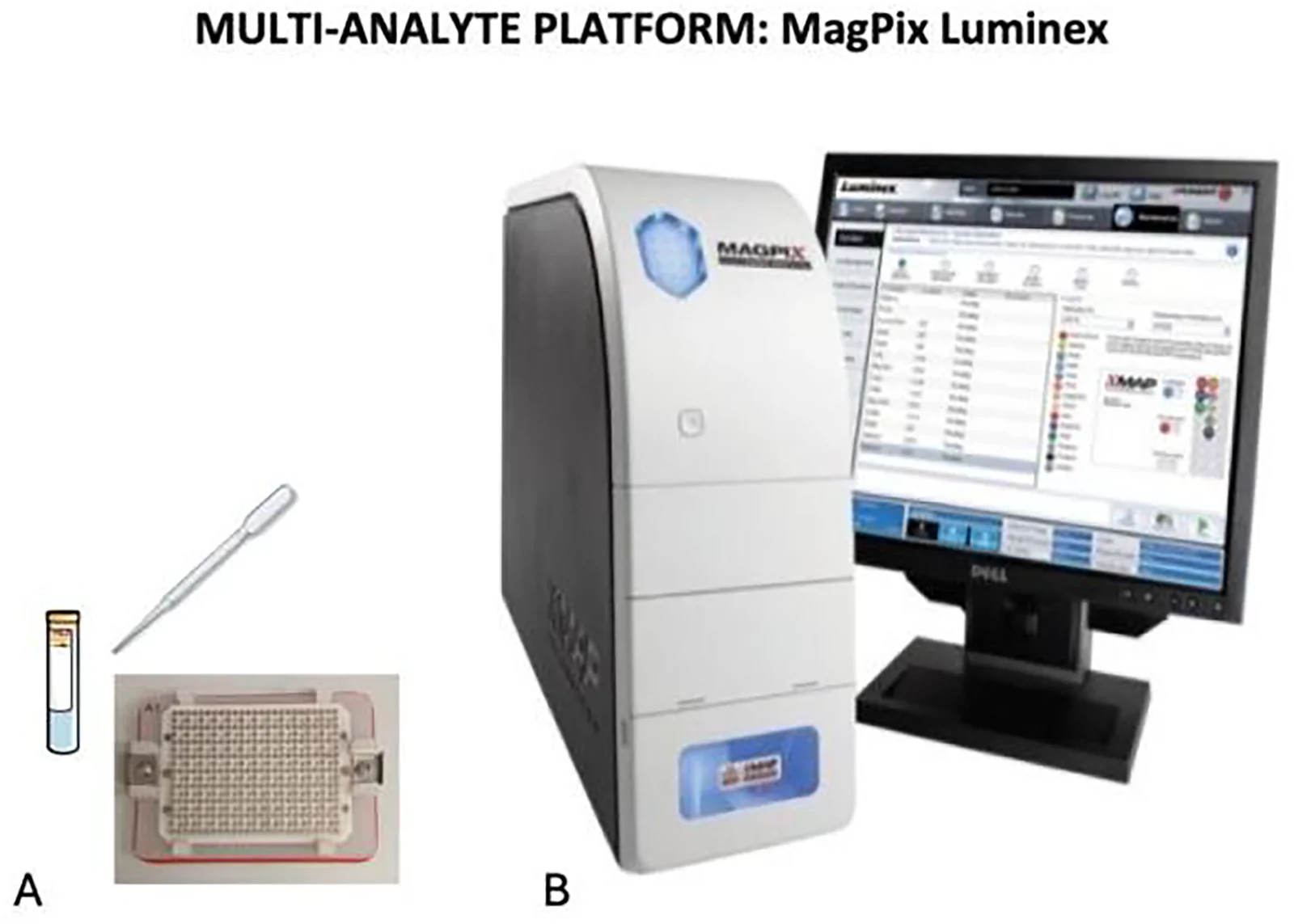
**(A)** Magnetic plate for fluorescent beads. A plasma sample is placed on the magnetic plate, where the magnetic beads are captured and react with the secondary antibody. **(B)** Magnetic bead reader and dedicated software for signal detection and result analysis.

RCII was calculated as the product of remnant cholesterol (RC, mg/dL, defined as the cholesterol content of TG-rich lipoproteins, obtained by subtracting HDL-C and LDL-C from TC) and hs-CRP (mg/L) divided by 10.

Follow-Up: Patients were followed during a mean follow-up period of 19 ± 4 months. Follow-up was recorded as follows: 1) review of the patient’s record, 2) telephone interview conducted by trained operators, 3) personal communication with the patient’s physician, 4) medical visit to the outpatient clinic. CV mortality was the clinical event considered and collected from medical records or death certificates (22). The definition of cardiac death required the following documentation: significant arrhythmias, cardiac arrest, death attributable to congestive heart failure, or myocardial infarction in the absence of any other precipitating factors.

## 3 Statistical methods

Continuous variables were given as mean ± SD, while categorical variables were reported using frequencies (percentages). The logarithmic transformation was used to make data closer to a normal distribution and stabilize variance (when distributions skewed to the right).

The comparison between two groups was assessed using Student’s t-test for continuous variables. Linear regression was performed to assess the relationship between a continuous dependent variable and one or more independent variables (predictors).

Cox proportional hazards regression was also performed as an exploratory analysis to investigate the association between RCII and cardiovascular mortality. Candidate variables included the anthropometric, clinical and biochemical variables available in the dataset and reported in **Table 1**, **2**. Variables showing an association with cardiovascular mortality in the preliminary Cox analyses were considered for the multivariable model.

**Table 1:** Anthropometric and clinical parameters of the patient population.

Parameter	Value
Number	103
Age (yrs)	83.5 ± 5.5
Sex (M/F)	33/70 (32/68)
Body mass index (kg/m^2^)	26.6 ± 4.7
Hypertension	91(88)
T2D	37 (36)
Dyslipidemia	76 (74)
Smoking habit	28 (27)
COPD	43(42)
Previous AMI	17 (17)
Previous stroke	13(13)
Chronic heart failure	66 (64)
Frailty (Fried criteria)	2.6 ± 1.3

Data are mean ± DS or number (%). T2D=type 2 diabetes; COPD=chronic obstructive pulmonary disease; AMI=acute myocardial infarction.

**Table 2:** Mean values of the biochemical parameters in the overall studied population.

Parameter	Value
Glucose (mg/dL)	116 ± 34.6
Total cholesterol (mg/dL)	185 ± 36.6
Triglycerides (mg/dL)	123.8 ± 67.4
HDL-C (mg/dL)	56.2 ± 15.7
LDL-C (mg/dL)	106 ± 29.3
CRP (mg/dL)	0.71 ± 1.4
ESR (mm/h)	38 ± 23
Fibrinogen (mg/dL)	377 ± 120.6
Uric acid (mg/dL)	6.1 ± 1.9
Neutrophils (10^3/µL)	5.1 ± 2
Lymphocytes (10^3/µL)	1.7 ± 0.9
Monocytes (10^3/µL)	0.6 ± 0.2
Platelets (10^3/µL)	226 ± 64
Creatinine (mg/dL)	1.4 ± 1.2
Albumin (g/dL)	4 ± 0.5
BNP (pg/mL)	571.5 ± 968.8

Data are mean ± DS.

HDL, high-density lipoproteins; LDL, low-density lipoproteins; hsCRP, high-sensitivity C reactive protein; ESR, erythrocyte sedimentation rate; BNP, brain natriuretic peptide.

A p value <0.05 was considered statistically significant.

## 4 Results

### 4.1 Clinical characteristic of patients

Clinical features of the patient population are reported in **Table 1**: the mean age was 83.5 ± 5.5 years and the majority of patients were females (68%). Hypertension was relieved in 88% of patients, T2D in 36% and dyslipidemia in 74%.

All patients presented a severe aortic stenosis with preserved systolic function at the echocardiographic assessment, that means all patients with an effective orifice area <1 cm^2^, presenting a mean EF 57.3 ± 8.8%, a mean mAVG corresponding to 44 ± 13 mmHg, a mean PAPs of 48.8 ± 11.4 mmHg. Aortic valve treatment included surgical aortic valve replacement (SAVR) in 7 (7%) patients, transcatheter aortic valve replacement (TAVR) in 56 (54%), balloon aortic valvuloplasty (BAV) in 26 (25%), and medical therapy (MT) in 14 (14%) patients.

Biochemical parameters are reported in **Table 2**. RCII was 19 ± 45 in the overall population; IL-6 and VEGF, obtained in a subgroup of patients (n=23), accounts for 3.5 ± 4.3 pg/mL and 32 ± 43 pg/mL, respectively.

### 4.2 Correlation between RCII and biochemical and inflammatory parameters

RCII showed a strong association with systemic inflammation, displaying a higher correlations with IL-6 (r=0.6; p<0.01, respectively) compared with neutrophils, neutrophils/lymphocytes, monocytes, platelets, ESR and fibrinogen (r=0.26, p<0.01; r=0.36, p<0.001; r=0.2, p<0.05; r=0.26, p<0.01; r=0.37, p<0.001; r=0.43, p<0.001, respectively). Significant positive correlations (even if weaker) were also found with glucose, creatinine (r=0.2, p<0.05; r=0.05, p=0.03, respectively). Moreover, a correlation between RCII with VEGF (r= 0.6; p<0.01; an inducer of endothelial cell growth) and with frailty (r=0.23; p=0.017). Inverse correlations were observed with albumin (r=-0.51; p<0.001, typically reduced in inflammatory states) and with HDL-C concentration (r=-0.33; p< 0.001).

### 4.3 Kaplan–Meier survival curve according to RCII levels and Cox proportional hazard models analysis for cardiovascular mortality

During the follow-up period (19 ± 4 months), 10 CV deaths were recorded. The Kaplan–Meier analysis showed that AoS patients with high RCII levels (>75^th^ percentile corresponding to 17.6) had a worse prognosis (**Figure 2**).

**Figure 2:**
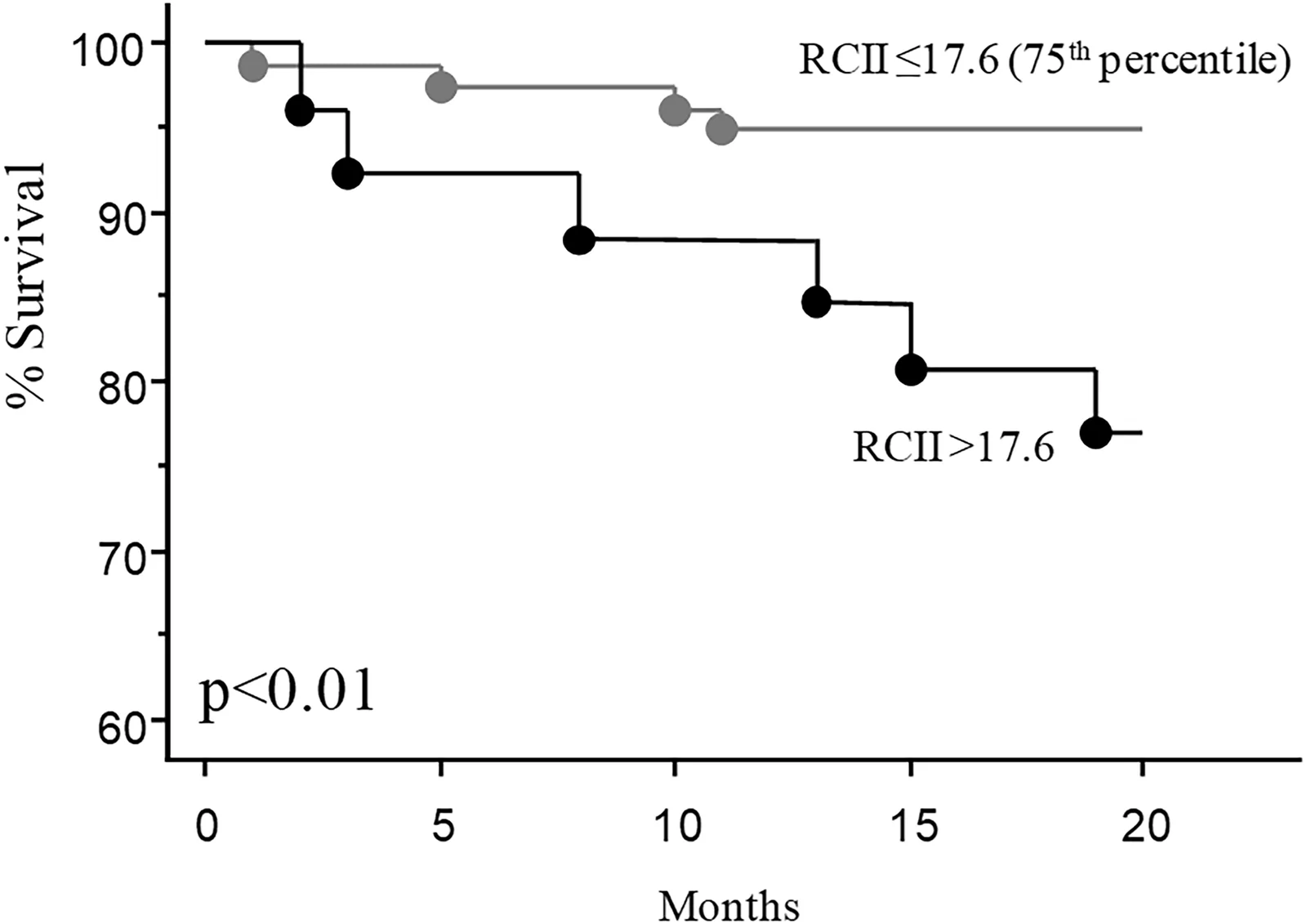
Kaplan-Mayer for mortality according to RCII values.

Values of the biochemical parameters were scored to be tested in the COX analysis according to laboratory reference values; age was scored according to 50th percentile (84 yrs), BMI according to standard classes (normal weight: BMI <24.9 kg/m²; overweight: BMI 25.0–29.9kg/m²; obesity >30 kg/m²). In our population, none of the anthropometric, clinical, or biochemical variables were associated with mortality in the Cox regression analysis, except for the variables reported in **Table 3**. RCII together with neutrophils remained as significant predictors for mortality after multivariate adjustment in this patient population (**Table 3**).

**Table 3:** Univariate and Multivariate Cox model for cardiovascular mortality.

	Univariate analysis			Multivariate analysis		
Predictor	HR	95% CI	p	HR	95% CI	p
RCII (>17.6; 75^th^ percentile)	4.7	1.3-16	<0.05	3.5	1-12	≤0.05
Neutrophils (>9.68 10^3/µL)	6.6	1.4-31	<0.05	5.1	1.1-24.7	<0.05
Fibrinogen (410> mg/dL)	4.2	1.2-14.9	<0.05	2.8	0.8-10.4	ns

95% CI, 95% confidence interval; HR, hazard ratio; p, p value.

## 5 Discussion

RCII was associated with inflammatory and cardiometabolic risk biomarkers as well as with frailty in our population of severe AoS older patients. In addition, exploratory survival analyses suggested an association between higher RCII levels and CV mortality. Given the limited number of CV deaths, however, this finding should be regarded as preliminary and hypothesis-generating rather than evidence of an independently established prognostic role. Overall, the present results highlight the complexity of the pathophysiological interplay among systemic metabolic dysfunction, frailty, inflammation, and mortality in elderly patients affected by severe degenerative AoS.

### 5.1 A possible underlying key mechanistic factor

Although not directly the focus of the present study, one key feature common to the above inter-relationship observed may be the NLRP3 inflammasome pathway. In fact, once activated, the NLRP3 complex activates caspase-1, with increased release of proinflammatory cytokine (e.g. IL-6) and induction of protein synthesis in the liver, which results in an acute phase pattern characterized by elevated levels of CRP, but also other biomarkers (e.g. fibrinogen). Lipids (e.g. ox-LDL) are important regulators of the NLRP3-inflammasome, which render NLRP3 inflammasome as a putative mechanistic link between vascular inflammation and the cholesterol pathway, underlying systemic atherosclerosis, aortic valve calcification and frailty (**Figure 3**).

**Figure 3:**
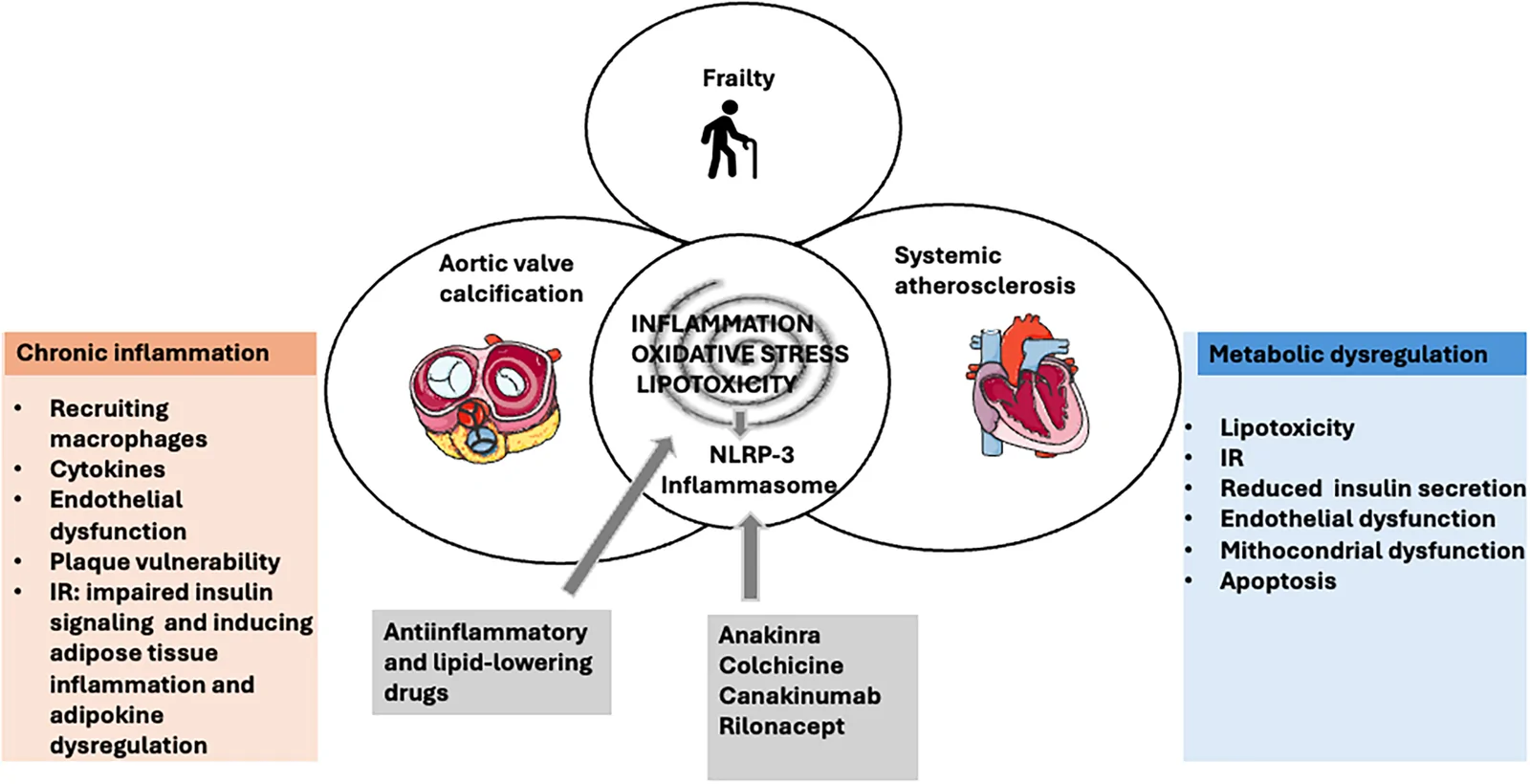
Biological mechanisms common to aortic valve calcification, frailty and systemic atherosclerosis and possible shared interventional strategies. IR, insulin resistance.

In fact, NLRP3 inflammasome activation may generate a vicious cycle amplifying the inflammatory milieu, which modulates the cellular recruitment and the onset of foam cells, eventually leading to the development of complex plaques in atherosclerosis (23). NLRP3 inflammasome also maintains chronic inflammation and favors osteogenic calcification AoS, promoting the transformation of valvular cells into bone-like cells, whereas its inhibition reduces or reverses the development of valve calcification in experimental models (24). Moreover, IL-18, one of the main products of NRLP3 inflammasome, has been identified as a mediator of aortic valve calcification, through promotion of the myofibroblast differentiation of valvular interstitial cells and calcification via the NF-κB pathway (25, 26). Interestingly, NLRP3 inflammasome has been also found related to frailty and physical disability (e.g. cachexia and sarcopenia) (27). In addition, very recent experimental data identify NLRP3 inflammasome activation as a key molecular correlate of cognitive frailty (28).

### 5.2 Inhibitors targeting NLRP3 inflammasome

Importantly, different inhibitors targeting NLRP3 inflammasome and downstream molecules (e.g. IL-1), such as anakinra (selective antagonist of IL-1 receptor prepared by gene recombination technology), colchicine (a common anti-inflammatory drug), canakinumab (monoclonal antibody that selectively inhibits IL-1β), rilonacept (IL-1 inhibitor) may represent a new, promising tool for early interventions to simultaneously counteract inflamma-aging, atherosclerosis and aortic valve calcification in elderly subjects (23). Although much work still needs to be done, this possibility represents an example of how molecular mechanisms can have a meaningful impact in clinical practice in a prospective of translational application (**Figure 4**).

**Figure 4:**
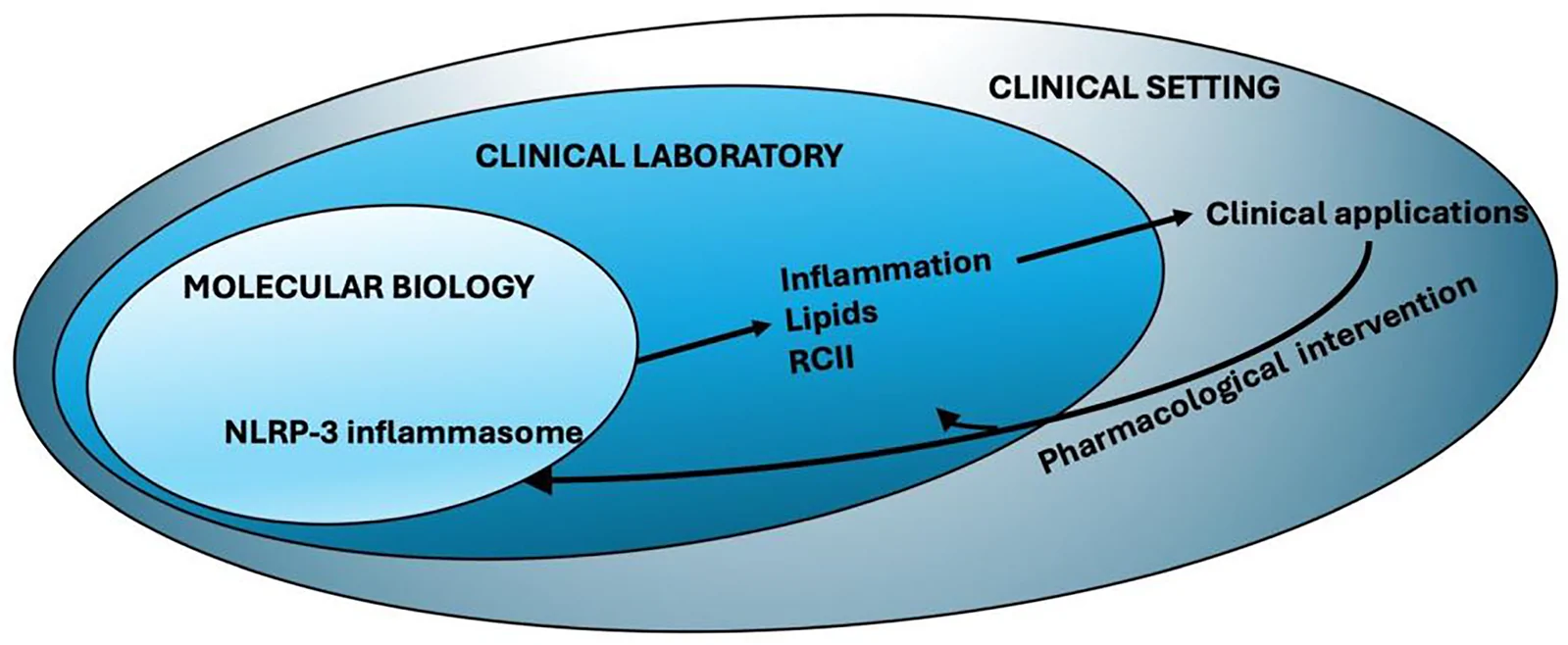
Interrelationship among molecular, laboratory and clinical settings in a prospective of translational application.

### 5.3 The properties of RCII derived from the joint capacity of RC and systemic low-grade inflammation

It is known that remnant particles are highly atherogenic and biologically active by infiltrating the arterial intima, activating vascular and immune cells, and contributing to endothelial dysfunction, vascular inflammation and cholesterol deposition (29, 30). Accordingly, high RC is related with an increased risk of CV disease, stroke (with higher levels linked to greater plaque vulnerability), and mortality (31, 32). Interestingly, elevated RC is also associated with an increased risk and progression of aortic valve stenosis (11). It is otherwise known that RC is associated with inflammation, insulin resistance (IR) and adverse metabolic profiles, contributing to the “residual risk” of T2D vascular complications: moreover, RC association with mortality seems to be modulated, almost in part, by its effect on IR (33–35). RC promotes lipotoxic injury to pancreatic β-cells, contributing to IR and diabetes development (36). This lipotoxicity may involve pathways common to RC and impairment of insulin secretion, such as endoplasmic reticulum stress and mitochondrial dysfunction, oxidative stress, and apoptosis (36, 37). In fact, RC correlates with surrogate and biochemical measures of IR, and IR partially mediates the RC–diabetes relationship (38). In the context of T2D, a recent meta-analysis conducted on 2,276,576 participants included in fifteen studies, evidenced an increased risk of CV events in T2D subjects with higher RC levels (39).

Complementarily, CRP exacerbates inflammation by activating the complement system and recruiting macrophages to arterial walls promoting production of cytokines, endothelial dysfunction, plaque vulnerability (40, 41). Moreover, CRP induces IR and T2D directly by impairing insulin signaling through the phosphorylation of insulin receptor substrates, but also indirectly inducing adipose tissue inflammation and adipokine dysregulation (42, 43).

Nonetheless, it is important to consider that in real life the lipid alterations (reflected by RC) and the systemic inflammatory state (represented by CRP) do not act independently, but rather together engage to promote the occurrence of adverse cardiometabolic events. Thus, inflammation represents both a consequence and an amplifier of RC-driven effects, through the RC induced cytokines and reactive oxygen species production by endothelial and innate immune cells, in a a loop in which remnant lipoproteins and inflammation may jointly affect IR and chronic disease pathogenesis. In different studies, the combined use of RC and CRP retains a better predictive value than using either indicator alone in the evaluation of CV risk (44, 45).

Accordingly, available data suggest that the combination of RC and CRP improved the predictive ability for severity of coronary artery disease (CAD) (46). High RC and CRP in combination correspond to a higher risk of CV disease outcomes (atherosclerosis, myocardial infarction, CAD and mortality) (44). In particular, high levels of CRP increased the risk of CV mortality associated with high RC (47). Accordingly, increasing data showed that the evaluation of RC and CRP in the RCII may jointly provide additive information beyond its individual components and a more comprehensive measure of risk. Although first results on RCII focused on stroke risk (5), subsequent studies expanded its application to all-cause and cause-specific mortality and as biomarker for more comprehensive risk stratification in a number of adverse cardiometabolic outcomes (48, 49). Recent data suggest that RCII predicts incident CV disease among patients in the early CV–kidney–metabolic stage (50). Other recent data indicated that in patients with acute decompensated heart failure, RCII represents a significant predictor of increased short-term mortality (51). Interestingly, in acute ischemic stroke (n=777), patients in the highest RCII quartile had almost double the risk of worse 3-month outcomes compared to those in the lowest quartile, with better discrimination for prognosis of RCII with respect to RC alone (52). Moreover, RCII appears related to increased risk of CV disease and some data suggest correlation between RCII and incident T2D, with increased metabolic syndrome risk in both US and Chinese general populations, and identify RCII as predictors of all-cause mortality and CV risks in individuals with preT2D or T2D (9, 10, 53, 54). Specifically, the finding that, in large U.S. and Chinese populations (3,743 NHANES and 9,616 CHARLS participants), subjects in the highest RCII quartile had almost sevenfold higher odds of metabolic syndrome is particularly noteworthy (55).

In our study RCII appears to be closely linked to the systemic inflammatory state. The strongest correlations are with CRP and IL-6, indicating that as the variable increases, inflammatory activation also increases significantly. This finding is further supported by the positive associations with ESR and fibrinogen, other classic markers of inflammation. Moreover a negative correlation is observed with albumin, which is typically reduced in inflammatory states or chronic disease conditions (55, 56). The lipid profile also shows a consistent trend since HDL is inversely correlated with RCII, suggesting an unfavorable metabolic profile associated with inflammation. Altogether, these results provide further evidence supporting RCII as a broad marker of inflammatory-metabolic burden, with the potential to improve risk stratification.

In the same time, other biomarkers emerge in this research area; in addition to progressive calcification, increasing evidence highlights the contribution of oxidative stress, chronic inflammation, lipoprotein deposition, and the activation of osteogenic signaling pathways to the pathogenesis and progression of AoS. Particularly attractive in the development of calcific aortic valve disease is nicotinamide adenine dinucleotide (NAD^+^), which levels decline with ageing. In particular, recent evidence suggests that calcific aortic valve disease may be initiated by endothelial NAD^+^ insufficiency and subsequently amplified by metabolically distinct macrophage populations, contributing to extracellular matrix inflammaging and accelerated valvular calcification (57). Moreover, Artificial Intelligence may help in this context to enable the identification of personalized biomarkers from the complex and concomitant mechanisms underlying senescence, degenerative, and metabolic cardiovascular diseases, thereby supporting their prevention and management.

### 5.4 RCII and VEGF

An additional interesting finding is the novel correlation between RCII and VEGF. VEGF is involved in AoS by mediating angiogenesis within calcified valves (58). Just in previous study authors highlighted the tissue coexistence of neoangiogenesis, T lymphocytes infiltration, adhesion molecules (VCAM, ICAM) and Hp60 gene expression, both in advanced calcific aortic valve stenosis as well as in calcific carotid plaque disease (1, 4), and increased s-VCAM-1 levels were found associated with calcific AS in patients with significant CAD (59). These data support an active endothelial function of tissue neovessels into an immunomediated inflammatory process. Moreover, VEGF overexpression promotes disease progression and increased blood VEGF values are related to advanced degenerative AoS in the elderly (60, 61). Unfortunately, VEGF was available for only a limited number of patients, as this parameter was not a primary focus of the study. Nevertheless, it certainly warrants further investigation in future studies.

### 5.5 RCII and frailty

Interestingly, we observed an association between RCII and frailty, an important determinant of survival in patients with AoS, that remains challenging to assess due to the decline of multiple physiological systems (19). Chronic inflammation, vascular endothelial dysfunction and altered glucose-lipid homeostasis are main culprits of frailty degree progression. Previous data also suggest a relationship between RCII and frailty (62). In fact, RCII, with its capacity to reflect all these processes, may represent a promising biomarker for identifying a multisystem vulnerability rather than a single disease entity, thus serving as a comprehensive metabolic risk indicator. Moreover, the dynamic interplay between lipid remnants and inflammation may be particularly interesting in the context of aging cohorts at high multisystem risk, where the coexistence of a multimorbidity with a subclinical organ impairment often antecede overt chronic structural diseases, frailty and negative clinical outcomes. Importantly, RCII is actionable, as lipid and inflammatory components could be modulated by using lipid-lowering and anti-inflammatory strategies or life-style changes. In this context, the use of statins in follow-up analyses over the past 2–3 years of major outcome trials, evidence a reduction of the atherosclerotic risk, related to high levels of remnants, where the benefit of high-dose versus low-dose of statin interventions are ascribed to the reduction in triglyceride-rich lipoproteins, RC or LDL-C levels (63). However, despite these similarities, therapeutic strategies effective in atherosclerosis, such as statin therapy, have not consistently shown benefits in AoS (64). Understanding the limits and potential of their overlap may inform future preventive and therapeutic strategies of new medical approaches aimed to delay aortic valve replacement and reduce the associated morbidity.

### 5.6 Limitations of the study

The retrospective nature of the study entails the inherent limitations associated with this type of study design.

IL-6 and VEGF were available in only 23 of the 103 patients, consequently results obtained cannot be considered representative of the complete cohort and need further validation; moreover, as these parameters were assessed in only a subgroup of patients, they were not included in the multivariate analysis.

Our data suggest that RCII may improve risk stratification, yet it does not demonstrate whether RCII adds predictive information beyond established clinical variables, including age, frailty, diabetes, renal function, BNP, aortic-valve gradient, previous myocardial infarction, and treatment strategy. An important limitation of the present study is the low number of cardiovascular deaths observed during follow-up (10 events among 103 patients), which limits the statistical power and precision of the survival analyses and increases the risk of model instability and overfitting in the multivariable Cox regression. The wide confidence intervals of the hazard-ratio estimates reflect this uncertainty. Therefore, the observed association between RCII and cardiovascular mortality should be considered exploratory and hypothesis-generating, without sufficient external validation or evidence of clinical decision impact to support routine stand-alone clinical use but supporting the hypothesis of a potential predictive value that requires confirmation in larger studies.

Although our findings suggest that RCII may contribute to risk stratification, the present study does not establish whether RCII provides additional predictive information beyond established clinical variables, including age, frailty, diabetes, renal function, BNP, aortic-valve gradient, previous myocardial infarction, and treatment strategy. For the same reason due to small sample, subgroup analyses according to sex, diabetes status, frailty, treatment strategy, or other clinical characteristics were not performed, as the limited sample size and number of CV events would have generated very small subgroups and potentially unstable estimates. Adequately powered studies should specifically investigate whether the association between RCII and CV outcomes differs across clinically relevant patient subgroups.

In conclusion, present results should be interpreted in light of the limited number of events and the consequent imprecision of the estimates and the observed association should be considered exploratory and hypothesis-generating rather than definitive. Moreover, the observed association warrants further investigation and confirmation in larger, prospective studies with adequate statistical power, external validation, standardized thresholds, serial measurements, and comparison with established risk prediction models before any consideration of clinical adoption. Nevertheless, we believe that the study provides clinically relevant preliminary data and addresses an important question that warrants further investigation.

## 6 Conclusion

Currently, no effective pharmacological treatments are available to prevent or delay the progression of aortic valve disease associated with aging. Although traditional markers of calcific AoS progression, such as increased transvalvular gradients and reduced aortic valve area, are routinely used to guide clinical decisions regarding valve replacement, these measures may not adequately capture the early stages of disease, when pathological changes may still be potentially modifiable. Therefore, there is a need for more sensitive biomarkers to monitor disease progression and potentially assess the efficacy of pharmacological interventions in calcific AoS (65). In this context, RCII integrates both metabolic and inflammatory pathways and, as a low-cost biomarker readily available from routine blood tests, it might be helpful in the clinical practice to widespread adoption at all levels in future. In fact, RCII might provide an additional perspective on the consequences of the co-occurrence of lipid abnormalities and low-grade inflammation in the elderly AoS population, potentially helpful to identify subjects at higher risk for earlier personalized preventive strategies (e.g. intensified lifestyle interventions or tailored pharmacotherapy). However, it still represents an exploratory biomarker (requiring external validation, standardized thresholds, serial measurement, and comparison with established risk models before clinical implementation) and its putative role warrants further evaluation in future prospective studies before its clinical implementation.

## Data Availability

The data would be available only for purpose researches as they represent sensible data of patients. Requests to access the datasets should be directed to Annamaria Mazzone, annamaria.mazzone59@gmail.com; Sergio Berti, berti@ftgm.it.
